# A chemical genetic strategy identify the PHOSTIN, a synthetic molecule that triggers phosphate starvation responses in *Arabidopsis thaliana*

**DOI:** 10.1111/nph.13591

**Published:** 2015-08-04

**Authors:** Clémence Bonnot, Benoît Pinson, Mathilde Clément, Stéphane Bernillon, Serge Chiarenza, Satomi Kanno, Natsuko Kobayashi, Etienne Delannoy, Tomoko M. Nakanishi, Laurent Nussaume, Thierry Desnos

**Affiliations:** ^1^ CEAInstitut de Biologie Environnementale et de Biotechnologie, Laboratoire de Biologie du Développement des PlantesSaint‐Paul‐lez‐DuranceF‐13108France; ^2^ CNRSUnité Mixte de Recherche 7265 Biologie Végétale & Microbiologie EnvironnementaleSaint‐Paul‐lez‐DuranceF‐13108France; ^3^ Aix‐Marseille UniversitéSaint‐Paul‐lez‐DuranceF‐13108France; ^4^ CNRSUnité Mixte de Recherche 5095 Institut de Biochimie et Génétique CellulairesBordeauxF‐33077 CedexFrance; ^5^ Université Bordeaux 2 Victor SegalenBordeauxF‐33000France; ^6^ INRAUnité Mixte de Recherche 1332 Biologie du Fruit et PathologieCentre INRA de BordeauxVillenave d'OrnonF‐33140France; ^7^ Metabolome Facility of Bordeaux Functional Genomics CentreIBVMCentre INRA de BordeauxVillenave d'OrnonF‐33140France; ^8^ Graduate School of Agricultural and Life Sciencesthe University of Tokyo1‐1‐1, YayoiBunkyo‐kuTokyo113‐8657Japan

**Keywords:** *Arabidopsis thaliana*, chemical genetics, *Oryza sativa*, phosphate homeostasis, phosphate starvation, PHOSTIN, *phr1;phl1* mutant

## Abstract

- Plants display numerous strategies to cope with phosphate (Pi)‐deficiency. Despite multiple genetic studies, the molecular mechanisms of low‐Pi‐signalling remain unknown. To validate the interest of chemical genetics to investigate this pathway we discovered and analysed the effects of PHOSTIN (PSN), a drug mimicking Pi‐starvation in Arabidopsis.
- We assessed the effects of PSN and structural analogues on the induction of Pi‐deficiency responses in mutants and wild‐type and followed their accumulation in plants organs by high pressure liquid chromotography (HPLC) or mass‐spectrophotometry.
- We show that PSN is cleaved in the growth medium, releasing its active motif (PSN11), which accumulates in plants roots. Despite the overaccumulation of Pi in the roots of treated plants, PSN11 elicits both local and systemic Pi‐starvation effects. Nevertheless, albeit that the transcriptional activation of low‐Pi genes by PSN11 is lost in the *phr1;phl1* double mutant, neither *PHO1* nor *PHO2* are required for PSN11 effects.
- The range of local and systemic responses to Pi‐starvation elicited, and their dependence on the PHR1/PHL1 function suggests that PSN11 affects an important and early step of Pi‐starvation signalling. Its independence from *PHO1* and *PHO2* suggest the existence of unknown pathway(s), showing the usefulness of PSN and chemical genetics to bring new elements to this field.

## Introduction

Phosphorus is a fundamental element for life. As for several other nutrients (nitrate, potassium, iron, etc.), spatial and temporal phosphate (Pi)‐deficiency triggers in plants local and long‐distance (systemic) molecular, biochemical and morphological responses, in order to adapt their physiology to the heterogeneous distribution and availability of Pi in soil.

Transcriptomic analysis in Arabidopsis has helped to decipher networks and clusters of target genes that are coordinately regulated by Pi‐starvation. For example, genes related to Pi‐homeostasis maintenance, involved in Pi absorption and acquisition, or in Pi retrieval are systemically upregulated. By contrast, genes locally induced are involved in stress responses (oxidative processes, defence responses and metal detoxification) (Thibaud *et al*., 2010).

There is accumulating evidence that these appropriate responses rely on sensing mechanisms and complex signalling cascades (see for review Chiou & Lin, 2011; Zhang *et al*., 2014). Local responses depend of the Pi concentration in the growth medium surrounding the tissues (Bates & Lynch, 1996; Svistoonoff *et al*., 2007; Thibaud *et al*., 2010), while systemic responses depend on the phosphorus status of the whole plant (Foehse & Jungk, 1983; Linkohr *et al*., 2002; Thibaud *et al*., 2010).

Because of their importance for plant acclimation to low‐Pi, numerous genetic studies have been done over the past 15 yr. Nevertheless, only few elements of these local and systemic signalling pathways have been unveiled (Yang & Finnegan, 2010; Abel, 2011).

Some transcription factors have been found controlling some of the Pi‐starvation responses. But only PHR1 and its paralogue PHL1 (PHR1‐like 1) regulate the expression of numerous genes involved in various responses induced by Pi‐starvation. Both are important for long‐distance Pi‐signalling regulating Pi transport and remobilization as well as the induction of anthocyanin biosynthesis and the changes in carbohydrate metabolism observed during Pi‐starvation (Rubio *et al*., 2001; Bustos *et al*., 2010). Interestingly, the loss of function of PHR1 also impairs some local responses to Pi‐starvation as the transcriptomic induction of the ribonuclease RNS1 (Rubio *et al*., 2001; Duan *et al*., 2008; Thibaud *et al*., 2010).

The regulation of PHO2, an ubiquitin‐conjugating E2 enzyme (UBC24) that tags for degradation of some proteins involved in Pi transport (Liu *et al*., 2012; Huang *et al*., 2013; Park *et al*., 2014), is by now the best described systemic signal linking leaves with roots that regulate Pi absorption (Fujii *et al*., 2005; Aung *et al*., 2006; Bari *et al*., 2006; Chiou *et al*., 2006). This regulation occurs through the action of the microRNA *miR399* that targets and cleaves PHO2 mRNA in the roots. The expression of *miR399* in the shoots depends on PHR1 and its transfer to the roots maintains an appropriate Pi‐homeostasis under Pi‐sufficient conditions. This shoot–roots movement makes of *miR399* the first component of a systemic signal specifically controlling Pi‐homeostasis.

Finally, the signalling steps responsible for the elicitation of the local responses to Pi‐starvation are unknown. However, in Arabidopsis, the inhibition of the primary root meristem activity when encountering a Pi‐deficient medium (Sánchez‐Calderón *et al*., 2005) was found to control the elicitation of the local transcriptional responses to low‐Pi (Lai *et al*., 2007; Thibaud *et al*., 2010).

Our knowledge on mechanisms governing local and systemic responses to low‐Pi is rudimentary. The connection between the known actors of these pathways remains obscure and the sensing mechanism(s) controlling the local and long‐distance responses to Pi‐deficiency are unknown. Functional redundancy supported by multigenic families and lethal mutations are known to be limiting elements when it comes to elucidate signalling pathways and could explain the difficulties encountered by genetics approaches to elucidate the Pi‐sensing mechanism.

Chemical genetics has been proved to be a powerful approach for studying biological processes and identifying relevant elements in signalling pathways in plants (Blackwell & Zhao, 2003; McCourt & Desveaux, 2010; Tóth & van der Hoorn, 2010). For example, the small molecule Pyrabactin was instrumental to discover the abscisic acid (ABA) receptors PYR1 (Park *et al*., 2009). Like genetic mutations, the small molecules (drugs) used in chemical genetic approaches can perturb biologic processes and thus represent tools to dissect any biological function. Furthermore, one drug can alter the activity of several proteins of the same family, thereby offering an alternative strategy to overcome higher plants functional redundancy in multigenic families. In addition, the drugs dose‐effects relation, allow the characterization of genes that display lethal loss of function (Cutler & McCourt, 2005). Finding and characterizing mutants (Zhao *et al*., 2003, 2007; Rojas‐Pierce *et al*., 2007; Park *et al*., 2009; Rosado *et al*., 2011) or natural accessions (Zhao *et al*., 2007) with altered sensitivity to the drug allows the discovery of genes that escaped the classical phenotype‐based genetics screens. For these reasons, we have considered chemical genetics as a new approach to study the low‐Pi‐signalling.

In this paper, we demonstrate that chemical genetics is a valid method to investigate Pi‐homeostasis regulation. We found that a screen of a chemical library can lead to the identification of a drug eliciting Pi‐starvation responses. The PHOSTIN (PSN) is a small organic compound that induces local and systemic Pi‐starvation responses, including Pi‐uptake and consequently uncoupling Pi‐content and Pi‐homeostasis regulation. We show that the transcriptional effects of PSN depend of PHR1 and PHL1. Therefore, PSN seems to interfere with an important and early regulatory step of phosphate sensing or signalling. Interestingly, the effect of PSN on the Pi‐content of treated plant was shown to be independent on PHO2 or PHO1 suggesting the existence of unknown signalling pathways. We identify the chemical motif responsible for PSN effects by characterization of the effects of PSN analogues and the measure of PSN and analogues content in treated plants. Our results demonstrate the interest of both PSN and chemical genetic as tools to dissect the Pi‐starvation pathway.

## Materials and Methods

### Plant material and general growth conditions

All *Arabidopsis thaliana* L. (Heynh.) lines used are in the Columbia (Col‐0) or in the Col^*er105*^ backgrounds, a Columbia background with the null allele *erecta‐105* (NASC reference N89504; Torii *et al*., 1996), except the *pPHT1;4::*β*‐glucuronidase* (*GUS*) line in WS background (Misson *et al*., 2004). Various Arabidopsis transgenic and mutant lines were used: *pIPS1::GUS* (Martín *et al*., 2000), *pSPX1::GUS and pSPX3::GUS* (Duan *et al*., 2008), *pPLD*ζ*2::GUS* (Cruz‐Ramírez *et al*., 2006), *pMGD3::GUS* (Kobayashi *et al*., 2009), *pSQD1::GUS* (Hammond *et al*., 2003), *pACP5::GUS* (Del Pozo *et al*., 1999), *pRNS1::GUS* (Hillwig *et al*., 2008), *pCHX17::GUS* (Cellier *et al*., 2004), *pSTP13::GUS* (Schofield *et al*., 2009) the mutant lines *pho1‐2* and *pho2‐1* (Delhaize & Randall, 1995), PHO1‐B1 (Rouached *et al*., 2011) and *phr1‐3;phl1‐2* (Bournier *et al*., 2013). Unless indicated, the Arabidopsis seedlings were grown as previously described (Svistoonoff *et al*., 2007).

The chemical library screen was performed in a modified liquid Murashige and Skoog medium (MS)/10 medium (+Pi medium) (0.15 mM MgSO_4_, 2.1 mM NH_4_NO_3_, 1.9 mM KNO_3_, 0.5 or 0.005 mM NaH_2_PO_4_, 0.34 mM CaCl_2_, 0.5 μM KI, 10 μM FeCl_2_, 10 μM H_3_BO_3_, 10 μM MnSO_4_, 3 μM ZnSO_4_, 0.1 μM CuSO_4_, 0.1 μM CoCl_2_, 1 μM Na_2_MoO_4_, vitamins: 5.9 μM thiamine, 4.9 μM pyridoxine, 8.1 μM nicotinic acid and 55 μM inositol in 3.4 mM MES (pH 5.8), 0.5% sucrose).

For the other *in vitro* analyses on Arabidopsis, seeds were grown on the previous medium without vitamins, supplemented with 0.8% agar.

For the low‐nitrate experiment, a nitrogen‐free MS/10 medium containing 75 μM of Pi was prepared, to which we added 50 μM KNO_3_ + 10 mM KCl or 10 mM KNO_3_ to make the low‐nitrate (−NO_3_^−^) and high‐nitrate (+NO_3_^−^) plates, respectively.

For the low‐sulphur experiment, a sulphur‐free MS/10 medium containing 75 μM of Pi was prepared to make the low‐sulphur (−SO_4_^2−^) medium, to which we added 2 mM MgSO_4_ to make the high‐sulphur plates (+SO_4_^2−^).

For the low‐potassium experiment, a potassium‐free MS/10 medium containing 75 μM of Pi was prepared to make the low‐potassium (−K^+^) medium, to which we added 1.9 mM KCl to make the high‐potassium plates (+K^+^).

Wild‐type seeds of *Oriza sativa* (L. cv Nipponbare) were cultivated on a KimuraB/2 medium (Tanoi *et al*., 2011).

For rice and Arabidopsis Pi‐deficient medium, an equivalent concentration of NaCl was used to replace the sodium provided by NaH_2_PO_4_.

For the split‐root experiments, the primary root tip of 5 d old after germination (5‐dag) seedlings was cut to induce the lateral roots growth. Four days later, plants were transferred in compartmented plates such that lateral roots lie on different media and grown 4 more days.

Drugs were dissolved in dimethyl sulfoxide (DMSO) and added to the growth medium before pouring the plates, and mocked controls were done with the same DMSO final concentration (+Pi DMSO).

### Chemical library screen and search for analogues

The Library of AcTive Compound on Arabidopsis (LATCA) (Zhao *et al*., 2007) was provided by Sean Cutler (UC Riverside) as 2.5 mM stock solutions dissolved in DMSO (Schreiber *et al*., 2008). Three seeds per well of Arabidopsis *pPHT1;4::GUS* lines were sown in 96‐wells plates containing 100 μl of MS/10 liquid medium. Five days after germination, drugs were added at 25 μM. After 2 d, the growth medium was replaced by the GUS staining solution and incubated overnight at 37°C. Plants were screened under a dissecting microscope. A secondary screen, performed in the same condition, on the first hits compounds (18 drugs) confirmed the activity of eight.

Structural analogues of the PSN were searched online in the Chembridge (http://chembridge.com) and Maybridge (http://www.maybridge.com) collections. Table 1 indicates their chemical formula, molecular weight, name, supplier and product code.

**Table 1:** Phostin (PSN) and PSN analogues Maybridge and Chembridge references

Name	Supplier	Product code	Molecular formula	MW	Product name
PSN	Maybridge	CD02621	C_19_H_14_ClN_3_O_5_	400	O5‐{[3‐(2‐chlorophenyl)‐5‐methylisoxazol‐4‐yl]carbonyl}‐1,3‐benzodioxole‐5‐carbohydroximamide
PSN2	Maybridge	SPB0273	C_16_H_13_ClN_4_O_3_S	377	O4‐{[3‐(2‐chlorophenyl)‐5‐methylisoxazol‐4‐yl]carbonyl}‐2‐methyl‐1,3‐thiazole‐4‐carbohydroximamide
PSN3	Maybridge	CD03092	C_16_H_16_ClN_3_O_3_	334	2‐[({[3‐(2‐chlorophenyl)‐5‐methyl‐4‐isoxazolyl]carbonyl}oxy)imino]piperidine
PSN4	Maybridge	CD02622	C_15_H_10_Cl_2_N_2_O_4_	353	O5‐(3,4‐dichlorobenzoyl)‐1,3‐benzodioxole‐5‐carbohydroximamide
PSN5	Maybridge	CD02598	C_8_H_8_N_2_O_3_	180	N′‐hydroxy‐1,3‐benzodioxole‐5‐carboximidamide
PSN6	Maybridge	SEW05933	C_13_H_12_ClN_3_O_3_	294	4‐chloro‐N′‐{[(3,5‐dimethylisoxazol‐4‐yl)carbonyl]oxy}benzenecarboximidamide
PSN7	Maybridge	SPB02369	C_18_H_13_Cl_2_N_3_O_3_	390	O1‐{[5‐(4‐chlorophenyl)‐3‐methylisoxazol‐4‐yl]carbonyl}‐4‐chlorobenzene‐1‐carbohydroximamide
PSN8	Chembridge	# 7991375	C_18_H_14_ClN_3_O_3_	356	N′‐({[3‐(2‐chlorophenyl)‐5‐methyl‐4‐isoxazolyl]carbonyl}oxy)benzenecarboximidamide
PSN9	Chembridge	# 5919370	C_18_H_13_ClN_2_O_4_	357	N‐1,3‐benzodioxol‐5‐yl‐3‐(2‐chlorophenyl)‐5‐methyl‐4‐isoxazolecarboxamide
PSN10	Chembridge	# 7984462	C_19_H_14_ClNO_5_	372	1,3‐benzodioxol‐5‐ylmethyl 3‐(2‐chlorophenyl)‐5‐methyl‐4‐isoxazolecarboxylate
PSN11	Maybridge	SB01885	C_11_H_8_ClNO_3_	238	3‐(2‐chlorophenyl)‐5‐methylisoxazole‐4‐carboxylic acid

### Microscopy and root length measurement

GUS activity was assayed as described (Sarrobert *et al*., 2000). Pictures were taken on a transmitted light microscope (DMRXA; Leica Microsystems, Wetzlar, Germany) (×10 or ×20 magnification) or on dissecting microscope (MZ16A, Leica Microsystems) (×6 for leaves, ×25 for roots) (Leica Microsystems). Ten plantlets per condition were imaged and the experiments were done in triplicate.

The primary root length was measured using the software ImageJ (US National Institutes of Health, Bethesda, MD, USA). The average lengths were calculated from three independent experiments on 15 plantlets.

### Gene expression analysis

Total RNA was extracted from 8 to 12 roots of 14 dag plantlets with the RNeasy Mini Kit (Qiagen, http://www.qiagen.com). RNAs were treated with DNase (TURBO DNase Ambion, http://www.ambion.com) and the cDNA were produced using the Superscript III reverse transcriptase (Superscript VILO cDNA synthesis kit, http://www.invitrogen.com). Quantitative real‐time reverse transcription (qRT)‐PCR were performed on a 480 LightCycler thermocycler (Roche) using the manufacturer's instructions with Light cycler 480sybr green I master (Roche). We used *ROC3* (AT2G16600) in Arabidopsis and *eEF‐1*α (AK061464) in rice (Jain 2006) as reference genes for normalization. Primers sequences are described in Table 2.

**Table 2:** List of the forward and reverse primers (5′ to 3′)

Gene	AGI	F	R
*Arabidopsis*			
*ACP5*	AT3G17790	TTTGACATAAGAGTTGCGAGATG	GTGAGCTTCAGAGATTTATAGAGCC
*AMT1.1*	AT4G13510	ACACTGTGGCCAGTTAGGCG	CCGTGGGGATGTCTTTGAGA
*HAK5*	AT4G13420	TCTCGTAGCGCTCCTCAAAT	GCTGTGTGGTTGGAAGTTCA
*IPS1*	AT3G09922	CGAAGCTTGCCAAAGGATAG	TGAAGACTGCAGAAGGCTGA
*LPR1*	AT1G23010	GCACCATCAAAACTTCGCAGAGATCG	CCGGGCTATGTCTACCATTGTCAC
*NRT2.1*	AT1G08090	AGTCGCTTGCACGTTACCTG	ACCCTCTGACTTGGCGTTCTC
*NRT2.4*	AT5G60770	CCGTCTTCTCCATGTCTTTC	CTGACCATTGAACATTGTGC
*PHO1‐H1*	AT1G68740	GCCACCACAAGACAGAGCCAACCAAGGC	AGCAATGCAGTGTGCAAGGAGGTGGT
*PHR1*	AT4G28610	GCTCTTTCACTACCGCCAAG	GTTCAGCAGCAACCTTCTCC
*PLD*ζ*2*	AT3G05630	TTTTGAAGCCGTTTCTTGCT	TGCATTGCTGGAGACAAAAG
*ROC3*	AT2G16600	TCGGTGAAAGCTTGATCCTT	ATCGTGATGGAGCTTTACGC
*SDI1*	AT5G48850	GTCAGAGCCAAACATGCTCA	ACGAGGACGGAAAGATTTGA
*SULTR1;1*	AT4G08620	GCGAGAGGAGCAAGAAAATG	TCACCACTGGTCCTGGATTT
Rice			
*eEF‐1*α	Os03g08020.1	TTTCACTCTTGGTGTGAAGCAGAT	GACTTCCTTCACGATTTCATCGTAA
*OsPT2*	Os03g05640.1	GACGAGACCGCCCAAGAAG	TTTTCAGTCACTCACGTCGAGAC

### Pi‐content and Pi influx

The Arabidopsis Pi‐content and Pi‐uptake experiments were assayed as previously (Misson *et al*., 2004).

Detection of ^33^Pi accumulation was performed with imaging plates as described (Kanno *et al*., 2012). The data were analysed using the Image Gauge software (Fujifilm, Tokyo, Japan). Preliminary experiments have shown that Pi‐uptake was linear at least from 30 min to 2 h (data not shown).

### Starch and anthocyanin assay

Anthocyanin were extracted from 10 plants and quantified as described (Ticconi *et al*., 2001). Starch was stained with Lugol (Sigma). These experiments were done three times independently on five biological replicates.

### Phosphatase activity

The acid phosphatases were extracted from 5 mg (FW) of roots of 12 dag plants grown the last 7 d on the medium to test. We measured the phosphatase activity of the extract with a p‐nitrophenylphosphate colorimetric method (Kolari & Sarjala, 1995). The extract activity for each condition was calculated from three technical measures of three biological replicates and the experiment was conducted three times.

### PHOSTIN extraction and detection

PSN were extracted from shoots or roots of 7–10 dag plants transferred at 5 dag for 1–72 h on +Pi medium containing 25 μM of PSN2 (+Pi PSN2), or 48 h on the described compartmented conditions. Experiments were done twice for the kinetics of accumulation and once for the compartmented plants. PSN‐like compound were extracted by hydroalcoholic extraction done as described for metabolites (Moing *et al*., 2004) with two minor modifications: extractions were done on fresh material (50 mg^−1^ sample, *c*. 30–40 plantlets); samples were extracted successively with 300 μl of ethanol 100%, ethanol 50% (v: v) and pure water at 80°C for 15 min. Supernatants were combined and centrifuged at 25 000 ***g*** for 10 min. The resulting supernatant was lyophilized for 24 h. Extracts were resuspended in 200 μl of pure water and centrifuged at 21 000 ***g*** before separation by HPLC on a C18 Acclaim PA2 column (Dionex) (2.1 × 150 mm, 3 μm).

For detection of PSN and its derivatives in the medium, chemicals were added at 25 μM in +Pi liquid medium in sterile condition. Media were incubated in the culture chamber (16 h 24°C : 8 h 21°C, light : dark) and reactions were stopped at indicated times (30 min, 3, 16, 24 or 48 h) by freezing in liquid nitrogen. Samples (10 μl) were directly injected in the HPLC after thawing.

For ultraviolet (UV) light detection, the medium component and the drugs were separated by HPLC as described (Lecoq *et al*., 2001). We identified the absorption peaks of the drugs with their UV signature (data not shown) by using an UV spectrophotometer (Ultimate 3000 VWD; Dionex, Sunnyvale, CA, USA) and an UV diode array detector (Ultimate 3000 RS; Dionex) between 220 and 340 nm.

For mass spectrometry detection, metabolites and PSN of the plant extracts were separated on an Ultimate 3000 HPLC system (Dionex). Solvents A and B were respectively H_2_O with 0.1% formic acid and acetonitrile. Flow rate was 0.25 ml min^−1^. Separation gradient was 0–5 min 97% A; 5–30 min from 97% A to 5% A; 30–35 min 5% A; 35–36 min from 5% A to 97% A; 36–51 min 97% A. Mass spectrometry detection was performed using a micrOTOF‐Q (Bruker Daltonics, Bremen, Germany). The mass spectrometer was equipped with an electrospray ionization probe operated in positive mode. Spray voltage was 4.5 kV. Mass range was 70–1500 Th and acquisition rate was 1 spectrum 2 s^−1^. MS spectra were calibrated using a 10 μM lithium formiate solution. Comparing retention time, accurate mass and fragments with the commercial drug standard identified PSN2 and PSN11. Elemental formulas were calculated using the generate molecular formula module of the Data Analysis software (Bruker Daltonics). Extracted ion chromatograms at *m/z* = 377.04 ± 0.3 Th and 238.02 ± 0.3 Th were used for quantification of PSN2 and PSN11, respectively.

UV and mass detection quantification were done by injection of standards (2.5–100 μM) for PSN and its analogues and peaks areas integration. Linear correlations were observed between peaks areas and drugs concentration (data not shown). Calculations of the measure and the technical error were done by injecting three times 100 μl of solution containing 500 nM, 1, 2.5, 5, 10, 25 and 50 μM of PSN and its analogues (data not shown).

## Results

### PHOSTIN induces Pi‐starvation responses

Pi‐starvation induces the expression of hundreds of genes involved in various adaptive responses to low‐Pi (Hammond *et al*., 2003; Misson *et al*., 2005; Morcuende *et al*., 2007; Thibaud *et al*., 2010). To find drugs altering the Pi‐starvation signalling we screened the LATCA chemical library (Zhao *et al*., 2007) for chemicals inducing several of the Arabidopsis Pi‐starvation responses tested. We chose the LATCA library because this medium‐size library of structurally diverse compounds contains only biologically active molecules and therefore potentially increasing the chance to find interesting drugs by screening < 4000 different molecules.

*PHT1;4* encodes an Arabidopsis high‐affinity Pi‐transporter (Nussaume *et al*., 2011) specifically and strongly induced in response to low‐Pi in the root epidermis and tip (Misson *et al*., 2004). Therefore, we used a *PHT1;4::GUS* Arabidopsis gene‐trap line (Misson *et al*., 2004; Hirsch *et al*., 2011) as a visual reporter tool to identify candidate elicitors of Pi‐starvation responses within the LATCA chemicals. The responding drugs were then tested on several transcriptional, physiologic and morphologic Arabidopsis responses to Pi‐starvation.

Less than 0.25% of the LATCA chemicals (eight over 3580) induced the expression of the *PHT1;4::GUS* marker (data not shown). Only one of them, that we named PHOSTIN (PHOsphate STarvation responses INductor), was found to stimulate the expression of all the low‐Pi markers tested (see later) and therefore selected.

**Figure 1:**
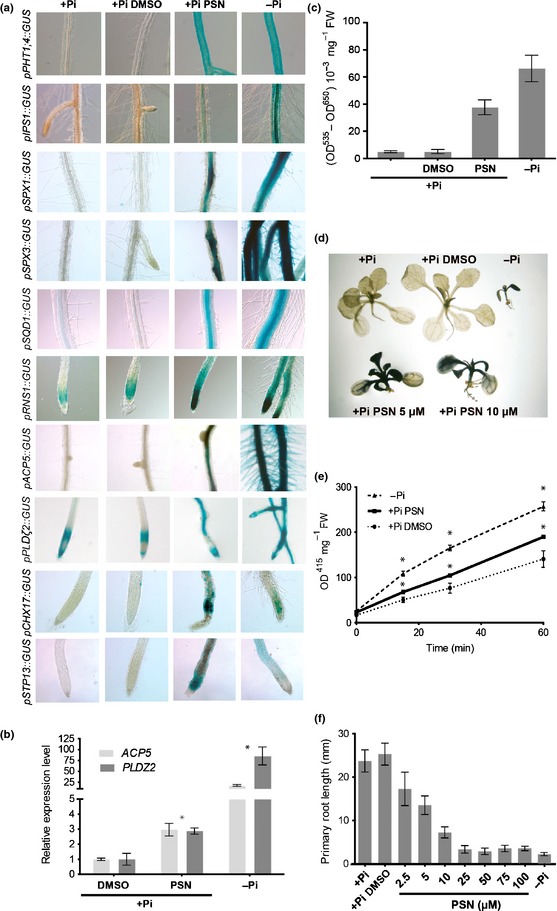
Phostin (PSN) induces phosphate starvation responses. (a) Effect of PSN on the expression of various phosphate (Pi)‐starvation reporter genes. (b) Relative expression of *ACP5* and *PLD*ζ*2*. Five days after germination wild‐type Arabidopsis seedlings were transferred on the indicated media for 4 d before total RNA extraction for quantitative real‐time reverse transcription‐PCR analysis. (c) Effect of PSN on anthocyanin accumulation. (d) Effect of PSN on starch accumulation. (e) Effect of PSN on phosphatase activity. (f) Primary root length of Arabidopsis seedlings grown in +Pi media. Seedlings were grown (a) 8 d, (c–e) 12 d or (f) 7 d in the indicated conditions. Growth conditions: +Pi; +Pi dimethyl sulfoxide (DMSO): (a, b) 0.25% DMSO or (d–f) 0.1% DMSO; +Pi PSN: (a, b) 25 μM, (d, e) 10 μM or (f) 2.5–100 μM and −Pi. For all quantitative experiments, averages ± SE were calculated from three independent experiments, except (b) (one experiment with two biological replicates). (b, e) *, Significantly different from +Pi DMSO treatment value (bilateral *t*‐test for sample of equal variance, *P* < 0.05).

As shown in Fig. 1(a), using GUS transcriptional reporter lines, the presence of PSN in a +Pi growth medium stimulates the expression of numerous genes that are known to respond to low‐Pi: *PHT1;4*,* IPS1*,* SPX1*,* SPX3*,* SQD1*,* RNS1*,* ACP5*,* PLD*ζ*2*,* CHX17 and STP13* (Essigmann *et al*., 1998; Martín *et al*., 2000; Gonzalez *et al*., 2005; Nakamura *et al*., 2005; Cruz‐Ramírez *et al*., 2006; Franco‐Zorrilla *et al*., 2007; Duan *et al*., 2008; Bayle *et al*., 2011). This observation was validated by the measure of the expression of the genes *ACP5* and *PLD*ζ*2* by qRT‐PCR (Fig. 1b).

PSN also stimulates typical physiologic responses to Pi‐starvation as the accumulation of starch and anthocyanin in leaves (Plaxton & Carswell, 1999; Wu *et al*., 2003; Misson *et al*., 2005) (Fig. 1c, d) or the root expression and secretion of phosphatases such as ACP5 (Duff *et al*., 1994; Köck *et al*., 1998; Trull & Deikman, 1998; Del Pozo *et al*., 1999; Haran *et al*., 2000). Indeed, in agreement with the higher expression of *ACP5* (Fig. 1b), the roots of PSN‐treated seedlings contain a phosphatase activity 1.35 times stronger than the mocked control (Fig. 1e). In addition, PSN has a similar inhibiting effect on the primary root growth as Pi‐starvation (Péret *et al*., 2011, 2014) (Fig. 1f).

These results show that PSN stimulates the expression of a large set of low‐Pi genes and induces typical physiological and morphological Pi‐starvation responses suggesting that PSN either affect the Pi‐starvation signal or homeostasis. Moreover, in rice, PSN treatment doubled the expression level of *OsPT2* (Ai *et al*., 2009), an homologue of the Arabidopsis *PHT1;4*, suggesting that the PSN target is conserved in different plant species (Supporting Information Fig. S1).

### PHOSTIN mimics the local and long‐distance Pi‐starvation regulations

As shown earlier, PSN induces the expression of genes known to be regulated either locally (*RNS1*,* STP13* and *CHX17*) or at long‐distance (*PHT1;4*,* IPS1*,* ACP5*,* PLDζ2*,* SQD1*) by Pi‐starvation (Thibaud *et al*., 2010). To test whether this induction by PSN follows the genuine local or systemic pattern of these genes, we performed a split‐root experiment in which we divided the root system of plants in two parts, each one fed with a different media.

**Figure 2:**
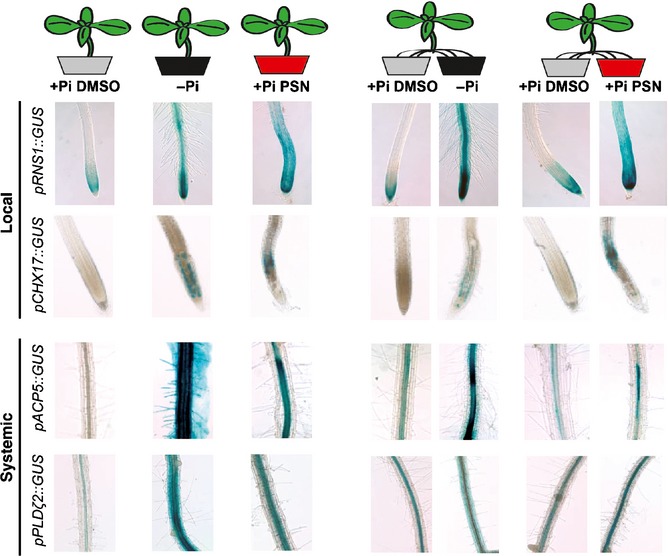
Phostin (PSN) treatment mimics local and systemic phosphate starvation regulation. Expression of genes locally regulated (*RNS1* and *CHX17*) or systemically regulated (*ACP5* and *PLD*ζ*2*) by phosphate (Pi)‐starvation thanks to the *promoter::β‐glucuronidase* (*GUS*) fusion Arabidopsis reporter lines: *pRNS1::GUS*,*pCHX17::GUS*,*pACP5::GUS* and *pPLD*ζ*2::GUS*. For control plants (left panel), lateral roots were grown on two identical media: +Pi dimethyl sulfoxide (DMSO)/+Pi DMSO (0.25% DMSO), −Pi/−Pi and +Pi PSN/+Pi PSN (25 μM PSN). For the other plants (right panel), lateral roots were grown on two different medium: +Pi DMSO/+Pi PSN or +Pi DMSO/−Pi.

We chose the *pRNS1::GUS* and *pCHX17::GUS* lines to test the effect of PSN on local induction and the *pACP5::GUS* and *pPLD*ζ*2::GUS* lines for PSN effect on the long‐distance induction (Fig. 2). In +Pi/−Pi split conditions, *RNS1* and *CHX17* are induced in roots lying on the −Pi medium but not in roots on the +Pi medium, as expected for local markers, whereas the long‐distance markers *ACP5* and *PLD*ζ*2* are induced in roots lying in both −Pi and +Pi medium. Interestingly in +Pi/+Pi +PSN split plants, PSN induces the same pattern of expression as does −Pi for these local and systemic genes. Remarkably, in split plants, the level of expression of the systemic genes is higher in roots directly in contact with PSN than in roots without. This pattern of expression is identical to the one observed in the +Pi/−Pi split control plants.

This striking result shows that PSN mimics both local and systemic effects of Pi‐starvation and therefore suggests that PSN affects an early step of the low‐Pi signal or Pi‐homeostasis.

### PHOSTIN uncouples Pi content and Pi‐starvation responses

**Figure 3:**
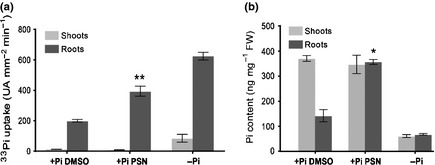
Phostin (PSN) increases phosphate uptake and accumulation. (a) Measure of ^33^phosphate (Pi) absorption and accumulation in PSN‐treated and untreated Arabidopsis Columbia (Col) seedlings in 15 min. **, ^33^Pi total absorption and ^33^Pi specific accumulation in roots are respectively significantly different from the ^33^Pi total absorption and root accumulation in untreated plants (+Pi dimethyl sulfoxide; DMSO) (bilateral *t*‐test for sample with unequal variance): *P *< 0.05. (b) Pi concentrations in shoots and roots. *, Significantly different from the Pi‐content in root of −Pi and +Pi DMSO plants (bilateral *t*‐test for sample with identical variance, *P* < 0.05). (a, b) Five days after germination wild‐type‐seedlings (Col) were transferred in the indicated growth conditions for seven more days before transfer on ^33^Pi medium: +Pi DMSO (0.1% DMSO); +Pi PSN (10 μM PSN) and −Pi. Averages ± SE for ^33^Pi uptake and Pi‐content were calculated from triplicates of three independent experiments.

The stimulation of Pi‐starvation responses in PSN‐treated plants could be the consequence of a reduced Pi‐uptake and content. To test the possibility that PSN represses Pi‐uptake, we measured the absorption of ^33^Pi in shoots and roots (Fig. 3a). Interestingly, PSN‐treated plants absorb ^33^Pi twice more rapidly than mocked plants (DMSO). These results corroborate the inductive effect of PSN on the expression of *PHT1;4* (Fig. 1a), a crucial transporter for Pi absorption (Misson *et al*., 2004; Shin *et al*., 2004; Ayadi *et al*., 2015).

Surprisingly, and by contrast to Pi‐starved plants, this increased absorption is limited to the root system. The speed of ^33^Pi accumulation in the shoots is identical in the PSN‐treated plant and mocked plant (Fig. 3a). This asymmetric effect of PSN on the speed of Pi absorption in shoots and roots is correlated to the Pi‐content of these tissues (Fig. 3b). Thus, although roots of PSN‐treated plants absorb more Pi than the untreated control, the amount of Pi transferred and stored in shoots is identical to the control plants. It seems therefore that PSN stimulates Pi‐uptake in the roots but does not increase Pi‐transfer to, or accumulation in the shoot.

These results show that PSN does not reduce the Pi‐content in plants but heightens it by increasing their Pi‐uptake. This effect is presumably a consequence of the induction of the expression of the genes involved in Pi absorption as the high‐affinity Pi‐transporters in PSN‐treated plants. Therefore, PSN artificially uncouples the signalling of Pi‐starvation from the actual Pi‐content in cells, possibly by targeting a central component of the Pi‐signalling.

### PHOSTIN effects are independent of PHO1 and PHO2

PHO1 and PHO2 are two proteins controlling the balance of Pi between roots and shoots. The *pho1* loss‐of‐function mutant over‐accumulates Pi in roots when grown on a Pi‐rich medium (Poirier *et al*., 1991; Rouached *et al*., 2011). By contrast, the *pho2* loss‐of‐function mutant transfers more Pi from the roots to the leaves, resulting in a decrease of the root Pi‐content and the overaccumulation of Pi in the shoot. To alter the shoot to root Pi‐balance PSN might depend on PHO1 and or on PHO2.

**Figure 4:**
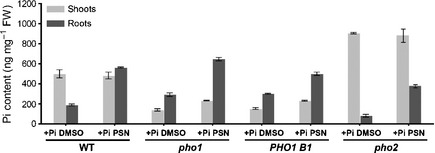
Effects of Phostin (PSN) on Pi accumulation in the *pho1* and *pho2* deficient lines. Five days after germination Arabidopsis seedlings were transferred on the indicated growth media. After 7 d, the free inorganic phosphate (Pi) concentration in roots and leaves was measured. Averages ± SE were calculated from triplicates of three independent experiments.

To test this idea, we measured the Pi‐contents in two *pho1* impaired lines (the *pho1* mutant and the *PHO1* under‐expresser *PHO1‐B1*), and in the *pho2* mutant (Delhaize & Randall, 1995; Rouached *et al*., 2011) treated with PSN. Unexpectedly, like in wild‐type (WT) plants, PSN increases the Pi‐content in both the shoot and roots of the *PHO1* deficient lines (Fig. 4). PSN also substantially reverts the reduced Pi‐content of *pho2* roots (Fig. 4).

These results show that PSN does not need PHO1 and PHO2 activities to modify the shoot to root Pi‐balance.

### The biological activity of the PHOSTIN relies on the hydrolytic release of its active motif PSN11

**Figure 5:**
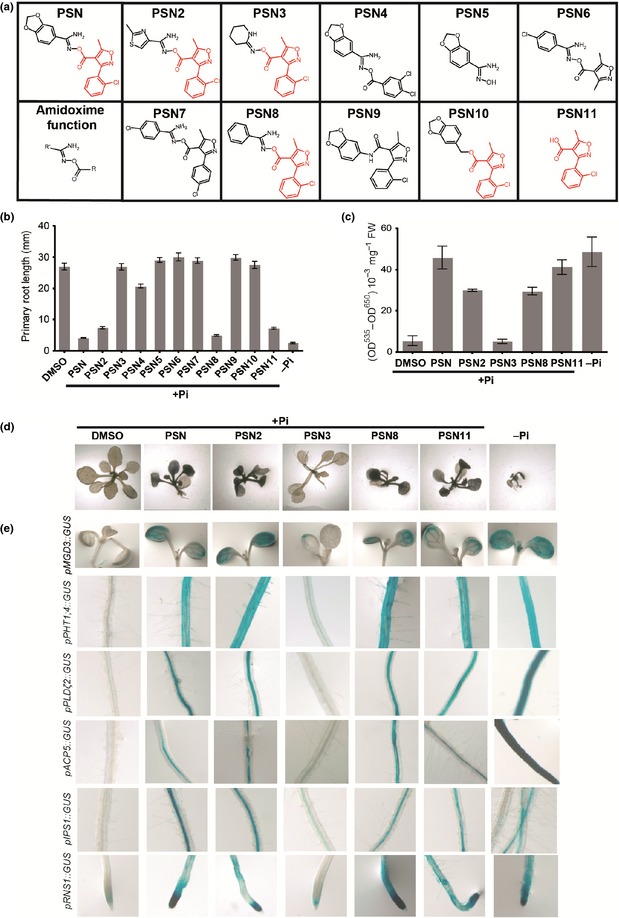
Structure–activity relationship of Phostin (PSN) structural analogues. (a) Chemical structure of PSN structural analogues. In red is drawn the motif corresponding to PSN11. (b) Primary root length of 8 d after germination (dag) Arabidopsis wild‐type seedlings treated with PSN and PSN analogues. (c) Anthocyanins accumulation in leaves of 12 dag seedlings. (d) Starch staining in 12 dag seedlings grown in presence of the indicated PSN analogues. (e) Expression of the indicated *promoter‐*β*‐glucuronidase* (*GUS*) fusion in seedlings. Plants were grown directly on the indicated medium: +phosphate (Pi) dimethyl sulfoxide (DMSO): (a, d) 0.1% or (b, c) 0.25%; +Pi supplemented with (a, d) 10 μM or (b, c) 25 μM of PSN or PSN analogues and −Pi. For (b, c) averages ± SE were calculated from 15 plants for each condition of three independent experiments.

A mean toward understanding PSN activity is to determine the active part of the molecule. For this purpose, we tested the effect of 10 structural analogues of PSN (PSN2 to PSN11, Fig. 5a) on WT plants.

Our results show that only three analogues (PSN2, PSN8 and PSN11) have a PSN‐like activity (i.e. induce all PSN responses as strongly as PSN) (Table 3). They repress the primary root growth (Figs 5b, S2a) and induce the accumulation of anthocyanin (Fig. 5c) and starch in leaves (Fig. 5d) of plants grown in +Pi. They also stimulate the expression of −Pi inducible genes by contrast to PSN3 taken as a negative control (Table 3; Figs 5e, S2b). Strikingly, these three actives (PSN‐like) analogues and PSN share a common structural motif corresponding to PSN11, the smallest analogue tested (Fig. 5a). Indeed, PSN11 is sufficient to display a PSN activity (Table 3). However, PSN3 and PSN10 also display the PSN11 motif (Fig. 5a), but they are not or poorly active in our biological assays (Fig. 5b,e; Table 3).

**Table 3:** Summary of the effects of the Phostin (PSN) analogues on the induction of phosphate (Pi)‐starvation markers in Arabidopsis seedlings

Drug	Primary root	Starch	Anthocyanin	*PHT1;4*	*IPS1*	*MGD3* Roots	*MGD3* Shoots	*PLDζ2*	*ACP5*	*RNS1*
DMSO	0	0	0	0	0	0	0	0	0	0
PSN	XXX	XXX	XX	XXX	X	XXX	X	XX	XX	XX
PSN2	XX	X	XX	XXX	XXX	XXX	X	XX	XX	XX
PSN3	0	0	0	XX	X	X	X	0	0	0
PSN4	0	0	0	X	X	0	0	0	0	0
PSN5	0	0	0	X	X	X	XX	0	X	0
PSN6	0	0	0	X	0	XX	XX	0	0	0
PSN7	0	0	0	X	X	X	0	0	0	0
PSN8	XXXX	XXX	XXX	XXX	XXX	XX	0	XXX	XXX	XX
PSN9	0	0	0	X	XX	X	0	0	X	0
PSN10	0	0	0	0	XX	X	X	0	0	0
PSN11	XX	X	XXXX	XXX	XX	XX	0	XXX	XX	XX

Effect of the PSN analogues (PSN2–11) treatments at 10 μM in +Pi medium were tested on the primary root growth inhibition (Primary root), starch accumulation in leaves (Starch), anthocyanin accumulation in leaves (Anthocyanin), induction of *PHT1;4*,* PHF1*,* IPS1*,* MGD3*,* PLD*ζ*2*,* ACP5* and *RNS1* expression in roots and induction of *MGD3* expression in shoots or roots. Gene expression was monitored with *promoter::*β*‐glucuronidase* (*GUS*) reporter lines. 0, no effect (+Pi 0.1% dimethyl sulfoxide (DMSO) treatment phenotype); X, low; XX, medium; XXX, high; XXXX, highest effect found for these drugs.

By comparing the structures of the active analogues with the inactive ones, we found that the PSN11 motif is covalently linked to an aromatic group by an amidoxime function in the active molecules (benzene in PSN and PSN8; thiazole in PSN2) (Fig. 5a). Amidoxime functions linking two aromatic cycles are prone to hydrolysis in acidic conditions (Clayden *et al*., 2001; see the scheme in Fig. S3a). Thus, by allowing the release of the PSN11, the hydrolysis of the amidoxime function provides a possible explanation for the biological activity of PSN, PSN2 and PSN8, and the absence of PSN‐like activity of PSN3 and PSN10 on seedlings in our growth conditions (pH 5.8).

To test this hypothesis we followed by HPLC‐UV spectrophotometry the evolution of the concentrations of PSN, PSN2, PSN3 or PSN11 when supplemented to our plant growth liquid media (Fig. S3b). In the medium supplemented with PSN11, the concentration of PSN11 remained stable. By contrast, in the media supplemented with PSN or PSN2, we observed a biphasic decrease of the concentration of these drugs (fast during the first 30 min and then slow), mirrored by a concomitant accumulation of PSN11. These kinetics show that PSN and PSN2 are unstable in our growth medium, and release PSN11. Note that PSN3 is also unstable but does not release PSN11.

We then tested the role of the pH of the growth medium on the hydrolytic release of PSN11 from PSN2. As shown in Fig. S3(c), the disappearance of PSN2 and the accumulation of PSN11 are faster at pH 5.0 than at pH 6.0, therefore confirming the acidic hydrolysis hypothesis. Finally, we tested the biological activity of PSN, PSN2, 8 and 11 at pH 5.0 and 6.0 (Fig. S3d). As measured on the repression of the primary root growth, the activity of PSN, PSN2 and PSN8 is higher at pH 5.0 than at pH 6.0, whereas the activity of PSN11 is identical at both pH.

Collectively these results support the view that the biological activity of the PSN‐like compounds relies on the hydrolytic release of the active chemical PSN11. The lack of an aromatic cycle linked to the amidoxime function in PSN3 (apart those of PSN11) and the lack of an amidoxime function in PSN10, prevent the release of PSN11 and explain their biological inactivity.

### The induction of systemic and local Pi‐starvation responses by PHOSTIN depends of the accumulation of PSN11 in the roots of treated plants

While PSN induces the systemic expression of long‐distance responses to low‐Pi, it does not promote at long distance the expression of the local response. This suggests that PSN11, the PSN bioactive derivative, does not move from the PSN‐treated roots to the untreated roots of split plants.

To know if and in which organ PSN11 and the PSN‐like drugs enters and accumulates, we used HPLC coupled with mass spectrometry. Among the PSN‐like drugs, PSN2 was the most stable in nonhydrolytic condition, for this reason plants were treated with PSN2 for different times and PSN2 and PSN11 were quantified in their shoot and roots separately.

**Figure 6:**
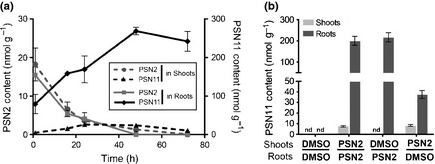
Phostin (PSN)11 enters and accumulates essentially in roots. (a) PSN2 and PSN11 content in plants. At 7 d after germination Arabidopsis wild‐type (WT)‐seedlings (Col) were transferred onto +phosphate (Pi) medium supplemented with 25 μM of PSN2. (b) PSN2 and PSN11 content in compartmented plants. At 7 dag WT‐seedlings (Col) were transferred onto compartmented plates such as the roots lies on another medium than the shoots. We used +Pi medium supplemented with 0.25% of dimethyl sulfoxide (DMSO) or 25 μM of PSN2 (PSN2). nd, not detected. For (a, b) PSN2 and PSN11 content were assayed in roots and shoots separately after (a) 1, 16, 24, 48 and 72 h of transfer or only after (b) 48 h of transfer by HPLC‐MS. Error bars, averages ± SD.

The concentration of PSN2 in roots and shoots of treated plants reaches its maximum 1 h after transfer but decreased to 25% of its maximum only 24 h after transfer and is undetectable 48 h after transfer (Fig. 6a). The kinetics of the decrease of the PSN2 in the plant resembles to the kinetics of its degradation in the medium (Fig. S3b, c).

By contrast, PSN11 progressively accumulates in the plant (Fig. 6a). Its concentration reaches a plateau *c*. 48 h after transfer, in both shoots and roots. The PSN11 concentration is 10 times higher in roots than in shoots already 24 h after transfer (Fig. 6a). All through the experiment, the concentration of PSN11 in roots is 5 to 10 times higher than the highest concentration of PSN2 in shoots or roots. The differences in the concentrations of PSN2 and PSN11 and their kinetic of accumulation in the plant reinforce the hypothesis that the active compound is PSN11 (or a PSN11 derivative).

To test whether PSN11 moves between root and shoot, we followed its accumulation in both organs of plants grown such that the roots and the shoot lie on different media. When only the roots of the plants are in contact with PSN2, we observe the accumulation of the PSN11 in roots but not in shoots (Fig. 6b). In addition, the PSN11 concentration in the roots of these plants is identical to the one of plants grown both roots and shoot in contact with the PSN2 medium. By contrast, when only the shoot lies on the PSN2 medium, accumulations of PSN11 is almost undetectable in the shoot and six times lower in roots compared with plants for which both shoots and roots were in contact with PSN2 (Fig. 6b). This indicates that PSN11 enters into the plants preferably by the root system, moves poorly from the shoot to roots and not in a detectable quantity from roots to the shoot or that PSN11 is quickly metabolized in the shoots.

**Figure 7:**
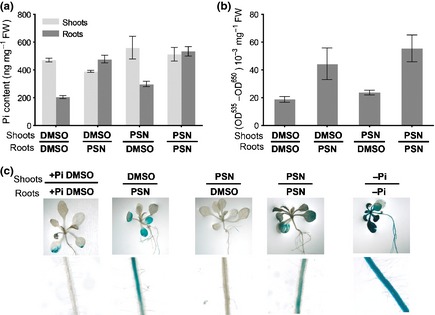
Phostin (PSN) effects depend on the root system. (a) Phosphate content in shoots and roots of compartmented Arabidopsis plants. (b) Anthocyanin content in shoots of compartmented plants. (c) *pMGD3::*β*‐glucuronidase* (*GUS*) expression in shoots and roots of compartmented plants. Five days after germination (a, b) wild‐type‐seedlings (Col) or (c) *pMGD3::GUS* were transferred onto compartmented plates such as the shoot and roots lie on different media: +phosphate (Pi), +Pi dimethyl sulfoxide (DMSO) (0.1% DMSO), +Pi PSN (10 μM PSN) and −Pi. After (c) 4 d or (a, b) 7 d plants were (c) GUS stained or harvested for (a) phosphate and (b) anthocyanin extraction and assay. For (a, b), averages ± SE for phosphate and anthocyanin were calculated from tree independent experiments with tree biological replicates per condition.

To test whether PSN11 accumulation in roots or putative metabolization by shoots have a prevalent role in PSN effects we monitored the induction of three PSN‐inducible responses (*MGD3* expression, increase of Pi and anthocyanin contents) when PSN is specifically delivered to the shoot or the roots. When only the root system is in contact with PSN the plant displays similar responses than when the whole plant is treated with PSN: roots accumulates as much as Pi (Fig. 7a), the anthocyanin content is increased in the shoot (Fig. 7b), and *MGD3* expression is induced in the whole plant (Fig. 7c). By contrast, when only the shoot lies on a PSN medium, the Pi‐content in roots is only slightly increased (Fig. 7a) and the anthocyanin content (Fig. 7b) and *MGD3* expression (Fig. 7c) are similar to the untreated controls.

In conclusion plants slightly respond to PSN when it is delivered to the shoot, whereas they strongly respond to PSN when it is supplied to the roots. This suggests that the root system is the major path of entry of PSN11 into the plant and that its direct contact with the drug is necessary for the PSN‐like effects.

### Transcriptional induction of low‐Pi markers by PSN11 depend of PHR1 and PHL1

**Figure 8:**
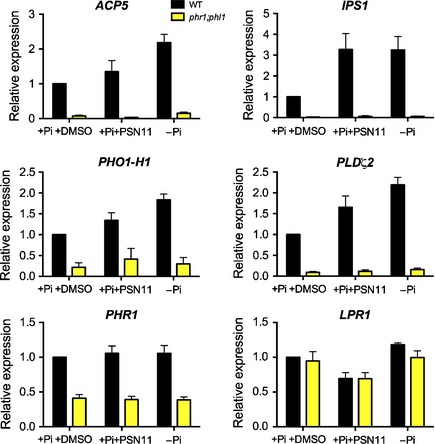
Relative expression level of genes related to the low‐Pi response. Five days after germination Arabidopsis wild‐type (WT) and *phr1‐3;phl1‐2* double mutant seedlings were transferred on the indicated media (−phosphate (Pi) = 0 μM Pi; +Pi = 75 μM Pi; + Phostin (PSN)11 = 25 μM PSN11; + dimethyl sulfoxide (DMSO) = 0.1% DMSO) for 4 d before root RNA extraction for quantitative real‐time reverse transcription‐PCR analysis. Error bars, averages ± SE from three independent experiments.

PSN‐likes drugs elicit effects on transcriptional, physiologic and morphologic, local or systemic low‐Pi Arabidopsis responses suggesting that PSN11 affect an early and important step of the low‐Pi‐signalling pathway. Only the paralogous transcription factors PHR1 and PHL1 are known to be involved in the regulation of numerous local and systemic responses to Pi‐starvation in Arabidopsis. To test if the PSN‐like drugs targets the PHR1 and PHL1 pathway we compared the effect of PSN11 on the expression of typical low‐Pi marker genes in roots of WT and double loss‐of‐function mutant *phr1;phl1* plants. As shown in Fig. 8, the low‐Pi induced genes *ACP5*,* IPS1*,* PHO1‐H1* and *PLD*ζ*2* are induced by PSN11 in WT plants grown in high‐Pi but not in the *phr1;phl1* plants. These results clearly show that the transcriptional effect of PSN11 relies on the intact master transcriptional regulators PHR1 and PHL1 and therefore that PSN11 probably elicit the low‐Pi‐signalling pathway upstream PHR1 and PHL1 functions. Note that *LPR1* is not induced by PSN11, showing that PSN, like Pi‐starvation, do not inhibit root growth by increasing transcriptional expression of *LPR1*.

## Discussion

### Chemical genetic has allowed us to find a drug affecting many aspects of the Pi‐starvation signalling

As a strategy complementary to the classical genetics in the elucidation of the molecular component of the Pi‐starvation signalling, we have tried chemical genetics. Indeed, this new, large scale pharmacological approach has been successful in the elucidation of important steps of plant signalling pathways such as the ABA perception (Park *et al*., 2009) or brassinosteroid signalling (De Rybel *et al*., 2009). Nevertheless, at the beginning of this work it was uncertain whether chemical genetics could be successfully applied to such an elusive signalling pathway underlying plant Pi‐homeostasis.

We show here the identification of PSN, the first small synthetic molecule mimicking Pi‐starvation in plants. PSN has the remarkable ability to mimic Pi‐starvation at several levels.

The PSN and the PSN‐like drugs interfere with morphologic (primary root growth inhibition), physiologic (increase of phosphatase activity and anthocyanin and starch accumulation in leaves) and transcriptional plant responses to Pi‐starvation and mimic the effect of Pi‐starvation on their regulation (Figs 1, 5, 8, S2). All the genes, which are systemically regulated by Pi‐deficiency, were found induced systemically by the PSN, while the local markers genes responded only locally (Fig. 2).

The activation of gene expression is observed for several typical molecular markers of Pi‐starvation and it is robust. As a matter of fact, these markers belong to different classes of responses (local and long‐distance) and different biochemical pathways (Pi‐transporters, Pi‐signalling, lipids synthesis, phosphatases). Importantly, the expressions of *NRT2;4* and *AMT1;1*, induced in the root during nitrogen deficiency, and of *SDI1* and *SULTR1;1*, induced in the root during sulphur deficiency (Howarth *et al*., 2009; Hubberten *et al*., 2012), are not affected by PSN11 (Fig. S4a,b). By contrast, the expressions of *NRT2;1*, another maker of nitrogen deficiency, and of *HAK5,* a marker of potassium deficiency (Ahn *et al*., 2004), known to be upregulated during Pi‐starvation (Lejay *et al*., 1999; Tian *et al*., 2009; Zheng *et al*., 2013; Misson *et al*., 2005; Shin *et al*., 2005; Lei *et al*., 2011) are induced by the presence of PSN11 in the medium (Fig. S4a,c). Therefore we conclude that PSN11 is not a general inducer of transcriptional responses to starvation.

### The active motif of PHOSTIN and PSN‐like molecules is PSN11

By characterizing the PSN and several of its structural analogues, we have shown that their activities depend on the release of the active motif, PSN11. This release is low pH‐dependent and occurs in our growth medium (pH 5.8), most probably by the hydrolysis of the amidoxime function linking PSN11 to the remaining part of the compounds (Figs 6, S3). Indeed, we also observed that the proclivity of PSN and PSN‐like molecules to release PSN11 at acidic pH determines their biological activity. Correlated with this, PSN11 accumulates in plants by contrast to the other PSN‐like compound tested.

### The effect of PHOSTIN on the local and systemic responses depends on PSN11 accumulation in root

The accumulation of PSN11 in roots appears responsible of most of the effects displayed by the PSN‐like analogues. The very low level of PSN11 detected in roots when PSN2 is only provided to the shoot suggests that the roots play a major role in PSN11 entry inside the plant and consequently in its activity. The presence of PSN11 in the roots of plants receiving PSN2 from their leaves, associated with their absence of PSN responses suggests the existence of a content threshold of PSN11 in roots necessary to activate these responses (Figs 6, 7).

Since PSN11 is detected in roots when supplied to the shoot (Fig. 6), PSN11 traffic from the shoots to the roots has to be considered. However, the opposite experiment indicates that PSN11 does not or poorly move from the root system to the shoot. We formally cannot exclude that PSN11 moves to the shoot where it is rapidly degraded or metabolized. Nevertheless, the PSN stimulation of local markers only in the treated root in split‐root experiments suggests that the active chemical does not move inside the plant, from root to root or remains below its active level. Consequently, the long‐distance induction of responses by the PSN‐like drugs is most probably not due to the accumulation of PSN11 in the untreated organs. This raises the question of the moving component: a chemical derivative of PSN11 or an endogenous Pi‐starvation signal that move from roots to the shoot? Despite several attempts, the detection of a molecule derived from PSN11 in extracts of PSN‐treated plants has failed (data not shown). Further work is necessary to identify the moving signal.

### PHOSTIN target a component of the Pi signalling pathway controlled by PHR1 and PHL1

PSN increases the Pi influx and consequently the Pi‐content in roots (Fig. 3). It is puzzling that PSN‐treated plants contain higher levels of Pi and express the low‐Pi markers. The expression of these markers is thus uncoupled from the internal Pi‐content, suggesting that PSN disrupts the activity of a mechanism allowing the plant to adjust its low‐Pi molecular and physiological responses to its Pi‐content. The chemical structure of PSN11 does not inform us about its molecular target, and we are not aware of natural compounds sharing the PSN11‐structure. Interestingly, in an opposite way than PSN treatment, the under‐expression of PHO1 (in the *PHO1‐B1* lines), encoding an SPX protein involved in the root to shoot Pi translocation, uncouples the low‐Pi responses from internal Pi‐content. Despite a reduced Pi‐content of the shoot as the *pho1* loss‐of‐function mutant, *PHO1‐B1* plants display a transcriptomic profile resembling to the one of WT plants grown on +Pi (Rouached *et al*., 2011). However, since the *pho1* mutant and *PHO1‐B1* lines still respond to PSN, the function of PHO1 is not essential for the PSN‐like activity (Fig. 4).

What could be the signalling pathway targeted by PSN11? The induction of local and systemic responses to Pi‐starvation by the PSN‐like drugs (Figs 1, 2, 5, 7) suggests that PSN11 affects an early and important step of the Pi‐starvation signalling or homeostasis pathways. Among the known systemic regulators of the −Pi responses only PHR1 and PHL1 regulates systemic as well as some local −Pi markers (Rubio *et al*., 2001; Thibaud *et al*., 2010). The insensitivity of the double loss‐of‐function mutant *phr1;phl1* to PSN11 shows that these two master regulatory genes are necessary for PSN transcriptional activation of low‐Pi genes (Fig. 8). This suggests that PSN11 targets an effector located upstream of PHR1 and PHL1 in the Pi‐starvation pathway. This is in contrast with the unaltered effect of PSN in the *pho2* mutant (Fig. 4). It seems therefore that the phenotype induced by PSN only needs a subset of the known low‐Pi‐signalling proteins and that its effect on Pi accumulation, at least, is independent on the PHO2 systemic pathway (this suggests the existence of additional pathways controlling Pi‐homeostasis during a Pi‐stress signal).

In addition, PSN stimulates the transcription of *PHT1;4* (in Arabidopsis) and *OsPT2* (in rice) encoding high‐affinity Pi‐transporters in two plant species belonging to different clades. Although preliminary, this result suggests a phylogenetic conservation of the molecular target of PSN on Pi‐homeostasis, further supporting the idea that the PSN target is crucial for Pi‐homeostasis in plants.

In conclusion, our discovery of PSN‐like drugs show that chemical genetics can be used to dissect the Pi‐starvation responses and signalling. These drugs seem to target an early step of the Pi‐starvation signal. Therefore, their use will help us to identify new components of the Pi‐starvation signalling pathway. This is complementary to another drug (Phosphatin) identified in our laboratory that alleviates low‐Pi response (Arnaud *et al*., 2014).

## Supporting information

Please note: Wiley Blackwell are not responsible for the content or functionality of any supporting information supplied by the authors. Any queries (other than missing material) should be directed to the *New Phytologist* Central Office.

**Fig. S1** Phostin (PSN) effect on the rice *OsPT2* expression.
**Fig. S2** Effects of Phostin (PSN) structural analogues on the induction of phosphate starvation markers.
**Fig. S3** Acidic hydrolysis release of Phostin (PSN)11 from the active PSN analogues.
**Fig. S4** Relative expression level of genes related to the low‐nitrate, low‐sulphur and low‐potassium responses.
