# Supplementary material for: A chemical genetic strategy identify the PHOSTIN, a synthetic molecule that triggers phosphate starvation responses in Arabidopsis thaliana
Source: New Phytol. 2015 Aug 4;209(1):161–76. doi: 10.1111/nph.13591 (PMC4737292; doi:10.1111/nph.13591)
Supplement: Supplementary file 1 — Fig. S1 Phostin (PSN) effect on the rice OsPT2 expression. Fig. S2 Effects of Phostin (PSN) structural analogues on the induction of phosphate starvation markers. Fig. S3 Acidic hydrolysis release of Phostin (PSN)11 from the active PSN analogues. Fig. S4 Relative expression level of genes related to the low‐nitrate, low‐sulphur and low‐potassium responses. [file NPH-209-161-s001.pdf]

New Phytologist Supporting Information Figs S1–S4

Article title: **A chemical genetic strategy identify the PHOSTIN, a synthetic molecule that triggers phosphate starvation responses in *Arabidopsis thaliana***

Authors: Clémence Bonnot, Benoît Pinson, Mathilde Clément, Stéphane Bernillon, Serge Chiarenza, Satomi Kanno, Natsuko Kobayashi, Etienne Delannoy, Tomoko M. Nakanishi, Laurent Nussaume and Thierry Desnos

Article acceptance date: 1 July 2015

The following Supporting Information is available for this article:

Fig. S1 Phostin (PSN) effect on the rice *OsPT2* expression.
Fig. S2 Effects of Phostin (PSN) structural analogues on the induction of phosphate starvation markers.
Fig. S3 Acidic hydrolysis release of Phostin (PSN)11 from the active PSN analogues.
Fig. S4 Relative expression level of genes related to the low-nitrate (A), low-sulphur (B) and low-potassium (C) responses.

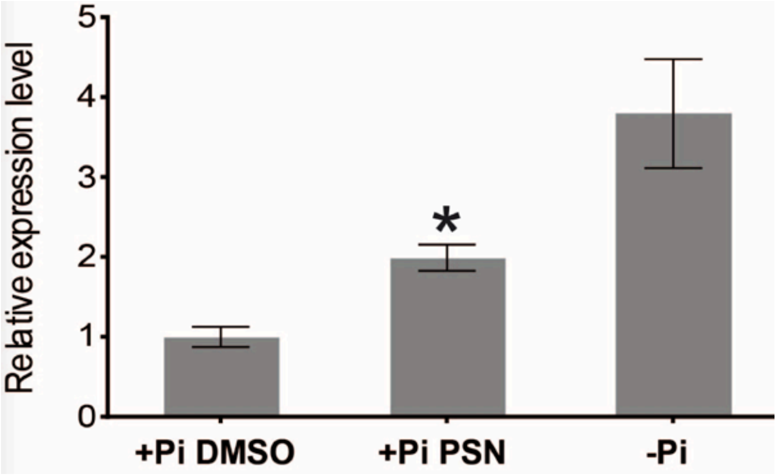

Fig. S1 Phostin (PSN) effect on the rice *OsPT2* expression. Relative expression of *OsPT2* measured by quantitative real-time reverse transcription (qRT)-PCR. *Oriza sativa* seedlings were grown 12 d in the indicated conditions: +phosphate (Pi): 91 µM Pi; -Pi: 0 µM Pi; dimethyl sulfoxide (DMSO): 0.1% DMSO; PSN: 10 µM PSN. Average ± SE were calculated from triplicates of three independent experiments. *, expression value significantly different from +Pi DMSO and -Pi (Bilateral *t*-test with unpaired sample with an equal variance).

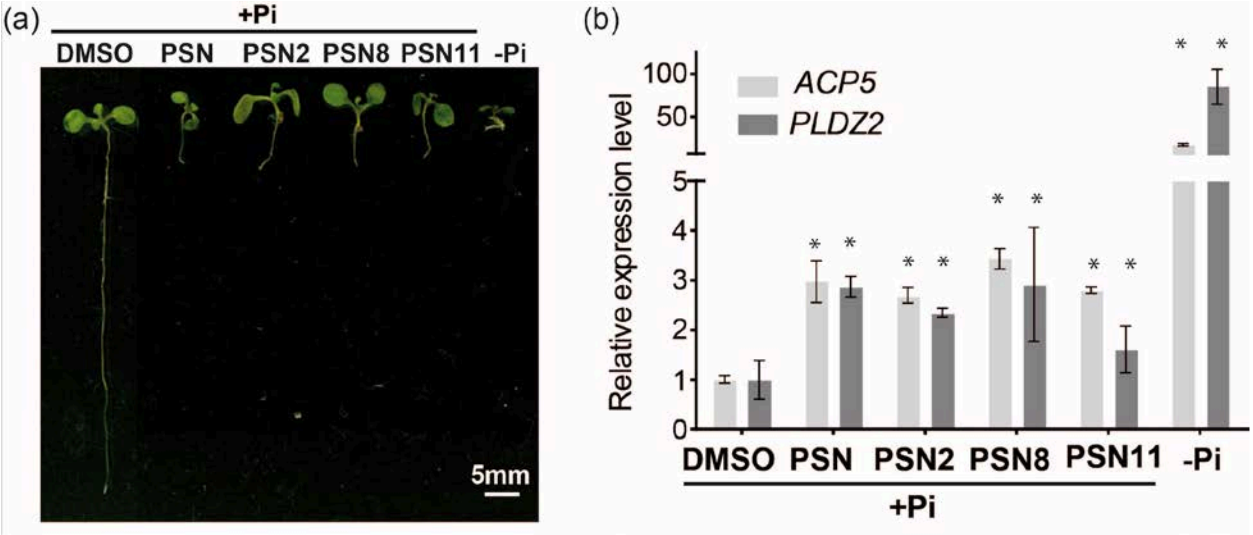

Fig. S2 Effects of Phostin (PSN) structural analogues on the induction of phosphate starvation markers. (a) Picture of seedlings grown with or without 10 µM of the indicated PSN analogues. (b) Relative expression of *ACP5* and *PLDζ2*. Five days after germination wild-type (Col) Arabidopsis seedlings were transferred in the indicated growth conditions for 4 d before extraction of RNA and quantitative real-time reverse transcription (qRT)-PCR analysis. Measures are expressed relatively to the +phosphate (Pi) dimethyl sulfoxide (DMSO) control. Growth conditions are: +Pi DMSO (0.25% DMSO), +Pi with 25 µM of PSN, PSN2, PSN8 or PSN11 and -Pi. Average ± SEM were calculated from three independent biological replicates. *, significantly different from +Pi DMSO treatment value (bilateral *t*-test for sample of equal variance) $P < 0.05$.

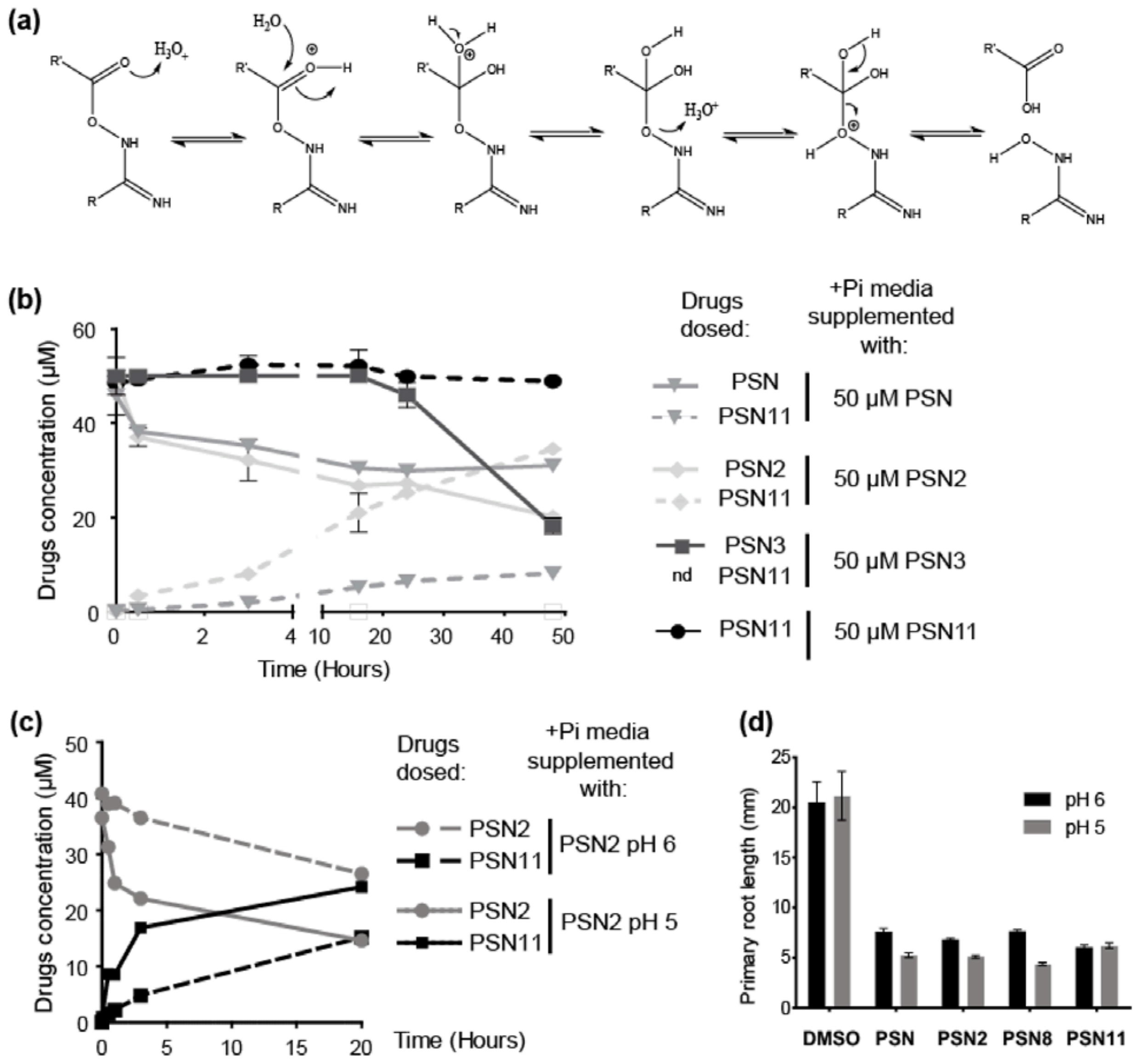

Fig. S3 Acidic hydrolysis release of Phostin (PSN)11 from the active PSN analogues. (a) Scheme of the acidic hydrolysis reaction of the amidoxine function. (b) Evolution of PSN, PSN2, PSN3 and PSN11 concentrations in the +phosphate (Pi) liquid growth media (pH 6) supplemented with 50 µM of PSN, PSN2, PSN3 or PSN11. The concentration of each compound was measured at 30 min, 3 h, 16 h, 24 h and 48 h after preparation of the media. Averages ± SE were calculated from triplicates and the experiment was independently duplicated. Nd, non-detected PSN11. (c) Evolution of PSN2 and PSN11 concentrations in the +Pi liquid growth media (pH 5.0 or 6.0) supplemented with 50 µM of PSN2. The concentrations were measured at 0 min, 30 min, 3 h and 20 h after preparation of the media. (d) Effect of the pH of the growth medium on

the activity of PSN analogues. Arabidopsis seedlings (Col) were grown 8 d on the indicated media : +Pi dimethyl sulfoxide (DMSO) (0.1% DMSO) or +Pi PSN or PSN analogue (10 µM) before root length measurement. Average ± SE were calculated from 15 plants per condition. The experiment was repeated independently three times.

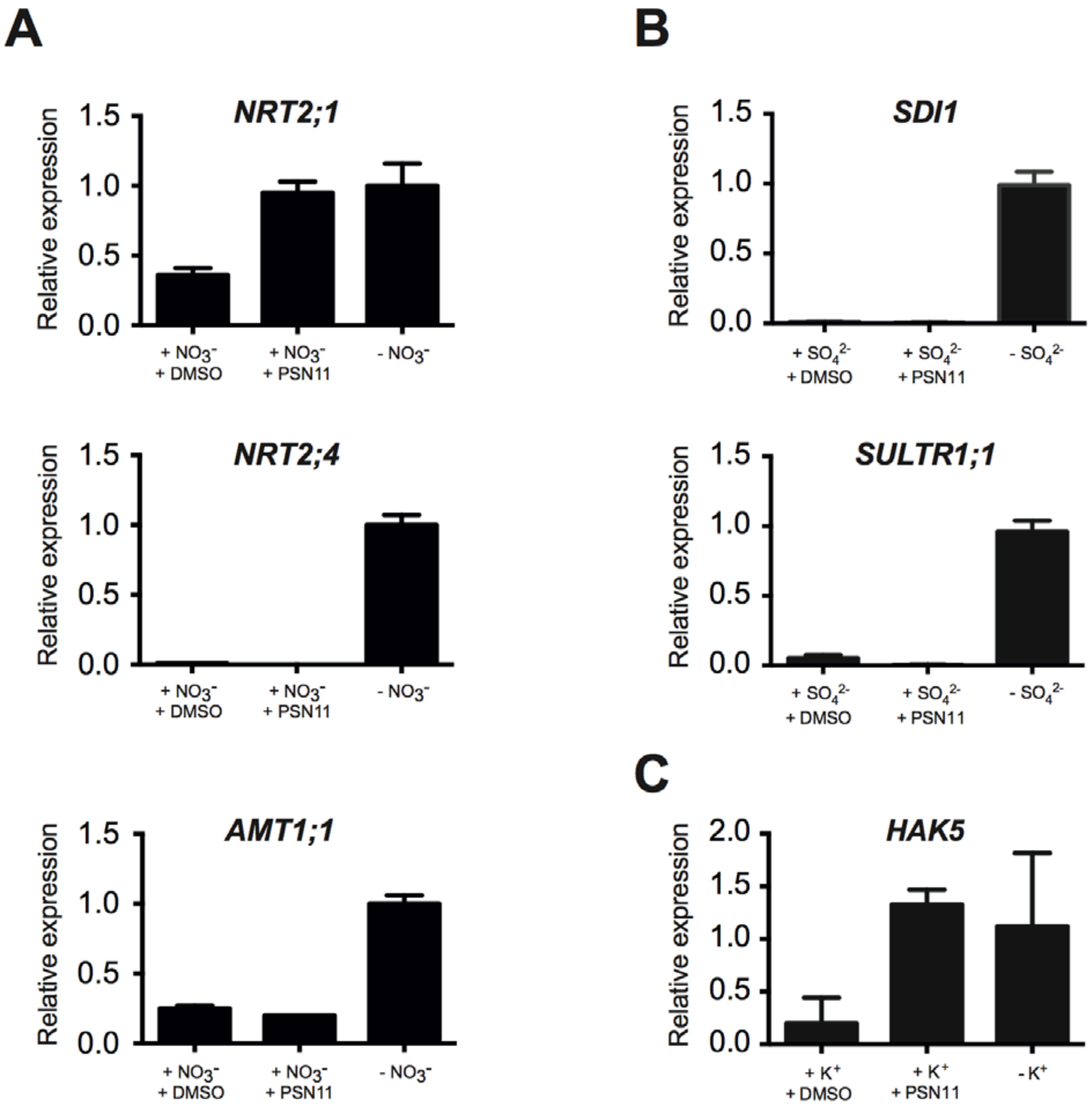

Fig. S4 Relative expression level of genes related to the (a) low-nitrate, (b) low-sulphur and (c) low-potassium responses. Five days after germination wild-type Arabidopsis seedlings were transferred on the indicated media (+Phostin (PSN)11 = 25 µM PSN11; + dimethyl sulfoxide (DMSO) = 0.1% DMSO) for 4 d before root RNA extraction for qRT-PCR analysis. Error bars, averages ± SE from three independent experiments.
